# Double-Vessel Involvement: A Case Report of Simultaneous Left Anterior Descending Artery (LAD) and Right Coronary Artery (RCA) Spontaneous Coronary Artery Dissection Causing Acute Coronary Syndrome

**DOI:** 10.7759/cureus.117104

**Published:** 2026-09-28

**Authors:** Fawad Qadir, Ali Hadi, Omar M Alhatou, Omar F Hassan, Haidar M Hadi

**Affiliations:** 1 Cardiology Department, Aisha Bint Hamad Al Attiyah Hospital, Hamad Medical Corporation, Doha, QAT; 2 Medicine, Royal College of Surgeons in Ireland, Busaiteen, BHR; 3 Internal Medicine, University of Northern British Columbia, Prince George, CAN

**Keywords:** acute coronary syndrome, conservative management, coronary angiography, ischemic cardiomyopathy, left ventricular thrombus, multivessel scad, spontaneous coronary artery dissection, subclavian artery occlusion, takayasu arteritis, vasculitis

## Abstract

Spontaneous coronary artery dissection (SCAD) is a rare cause of acute coronary syndrome (ACS). Here, we describe a case of multivessel SCAD in a 48-year-old Indian male who presented with ACS. His echocardiography confirmed a picture of moderate ischemic cardiomyopathy complicated by the presence of an apical clot, and coronary angiography confirmed SCAD in both the left anterior descending artery (LAD) and right coronary artery (RCA). He was also found to have an occlusion of the subclavian artery, raising the possibility of vasculitis, including Takayasu arteritis. The patient was managed conservatively with guideline-directed medical therapy for ACS and heart failure, along with anticoagulation for the left ventricular (LV) apical thrombus. He remained hemodynamically stable without recurrent ischemia or arrhythmias and was discharged in stable condition. This case highlights the importance of considering SCAD in ACS presentations and evaluating for associated arteriopathies or vasculitis in patients with multivessel involvement.

## Introduction

Spontaneous coronary artery dissection (SCAD) is a rare cause of acute coronary syndrome (ACS), accounting for 1-4% of cases, mainly among women [1]. Unlike atherosclerotic ACS, which results from plaque rupture and superimposed thrombosis, SCAD arises from a spontaneous, non-atherosclerotic separation of the coronary arterial wall, often in younger patients without traditional cardiovascular risk factors; recognizing this distinction is clinically important, as it changes both the diagnostic approach and the treatment strategy, since revascularization techniques routinely used in atherosclerotic disease may be unnecessary or even harmful in SCAD. Multivessel SCAD is considerably less common, being reported in approximately 9-23% of all documented SCAD cases [2]. Clinical manifestations vary widely, ranging from asymptomatic cases to unstable angina, acute myocardial infarction (MI), ventricular arrhythmias, or sudden cardiac death (SCD) [3]. Predisposing factors consist of fibromuscular dysplasia, pregnancy and the postpartum period, multiparity, mechanical stressors, emotional stress, connective tissue disorders (including Marfan, Ehlers-Danlos, and Loeys-Dietz syndromes), and systemic inflammatory diseases like vasculitis [4]. Management is usually conservative, but intervention may be necessary in selected cases, depending on the patient's hemodynamic stability and extent of coronary involvement [5]. Multivessel SCAD is particularly rare in male patients, and associated arteriopathies such as subclavian artery occlusion have been described only in a few reports, making this case noteworthy. Understanding such presentations is important for timely diagnosis and appropriate management.

## Case presentation

We present the case of a 48-year-old Indian male with no traditional cardiovascular risk factors (no diabetes, hypertension, dyslipidemia, smoking, or family history of coronary artery disease) and no identifiable predisposing factors for SCAD (no connective tissue disorder, recent pregnancy, extreme physical or emotional stress, or family history of SCAD), who presented in our emergency room with a complaint of chest pain for two days. The pain was moderate in intensity, retrosternal in position, dull to burning in nature, and radiating to the left arm. There were no other associated symptoms.

On examination, he was hemodynamically stable with the following vital signs: oral temperature 36.8°C, peripheral heart rate 92 beats per minute in normal sinus rhythm, blood pressure 113/86 mmHg, and peripheral oxygen saturation of 98 on room air. His cardiopulmonary examination revealed a clear chest with normal heart sounds and non-elevated jugular venous pressure. The rest of the examination was unremarkable.

His electrocardiogram (ECG) (Figure 1) revealed sinus rhythm with ST-segment elevation in V2-V4 with T-wave inversion and Q-waves. There was also T-wave inversion in leads I and aVL. Moreover, there was T-wave inversion with Q-waves in the inferior leads. These ECG findings were consistent on serial tracings, indicating an evolved myocardial infarction. His laboratory investigations on admission are summarized in Table 1, with serial troponin I levels showing a rising trend and peaking at 1095 ng/mL.

**Figure 1:**
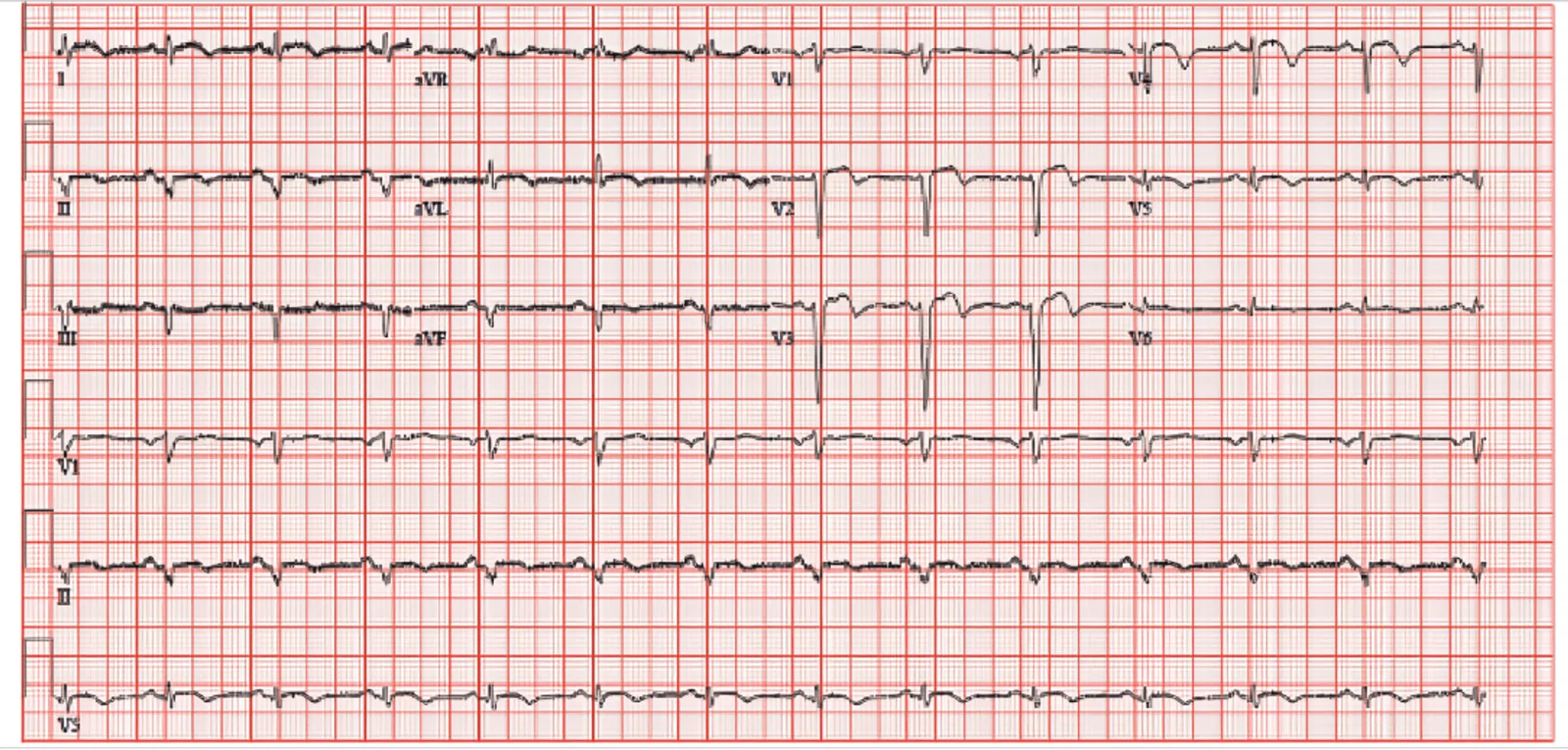
ECG showing evolved anterior wall myocardial infarction (MI) with Q-waves in the inferior leads.

**Table 1:** Laboratory investigations and findings Abbreviations: TC, total cholesterol; TG, triglycerides; LDL-C, low-density lipoprotein cholesterol; HDL-C, high-density lipoprotein cholesterol; PT, prothrombin time; APTT, activated partial thromboplastin time; INR, international normalized ratio; CRP, C-reactive protein; ESR, erythrocyte sedimentation rate

Laboratory test	Patient result	Normal reference range
Hemoglobin (g/dL)	16.4	13.5–17.5
Troponin I (ng/mL)	600 / 668 / 1095	<0.04
Urea (mmol/L)	4.4	2.5–7.1
Creatinine (µmol/L)	80	60–110
TC (mmol/L)	3.8	<5.2
TG (mmol/L)	1.1	<1.7
LDL-C (mmol/L)	1.8	<3.0
HDL-C (mmol/L)	1.1	>1.0
PT (seconds)	10.4	10–13
APTT (seconds)	29	25–35
INR	1	0.9–1.2
CRP (mg/L)	Within normal limits	<5
ESR (mm/hr)	Within normal limits	<20

He was diagnosed with a case of anterior wall MI, late presentation, and treatment of ACS was started, including intravenous heparin due to the presence of left ventricular (LV) apical thrombus. He underwent transthoracic echocardiography on the day of admission, which showed a normally sized left ventricle with moderately reduced systolic LV function. Biplane left ventricular ejection fraction (LVEF) was calculated at 39%, and global longitudinal strain (GLS) was -8% (abnormal: greater than -16%). Hypokinesia was observed in the basal anterior, basal anteroseptal, basal inferoseptal, mid anterior, mid anteroseptal, and mid inferoseptal segments of the LV wall. The basal inferior, mid inferior, apical anterior, apical septal, apical inferior, apical lateral, and apex segments were akinetic. Despite extensive regional wall motion abnormalities, the calculated LVEF of 39% is plausible due to preserved contractility in the basal segments, as reflected in the hypokinetic (rather than akinetic) status of those regions. There was grade 1 diastolic dysfunction (normal left atrial pressure). Focal wall thinning was not specifically commented on in the echocardiographic report; however, the extensive akinetic segments, in the context of late-presenting myocardial infarction with fixed Q-wave changes on ECG, are consistent with established myocardial scar rather than acute stunning. A large apical thrombus measuring 1.8 x 1.3 cm was identified with the use of contrast at the LV apex (Figure 2).

**Figure 2:**
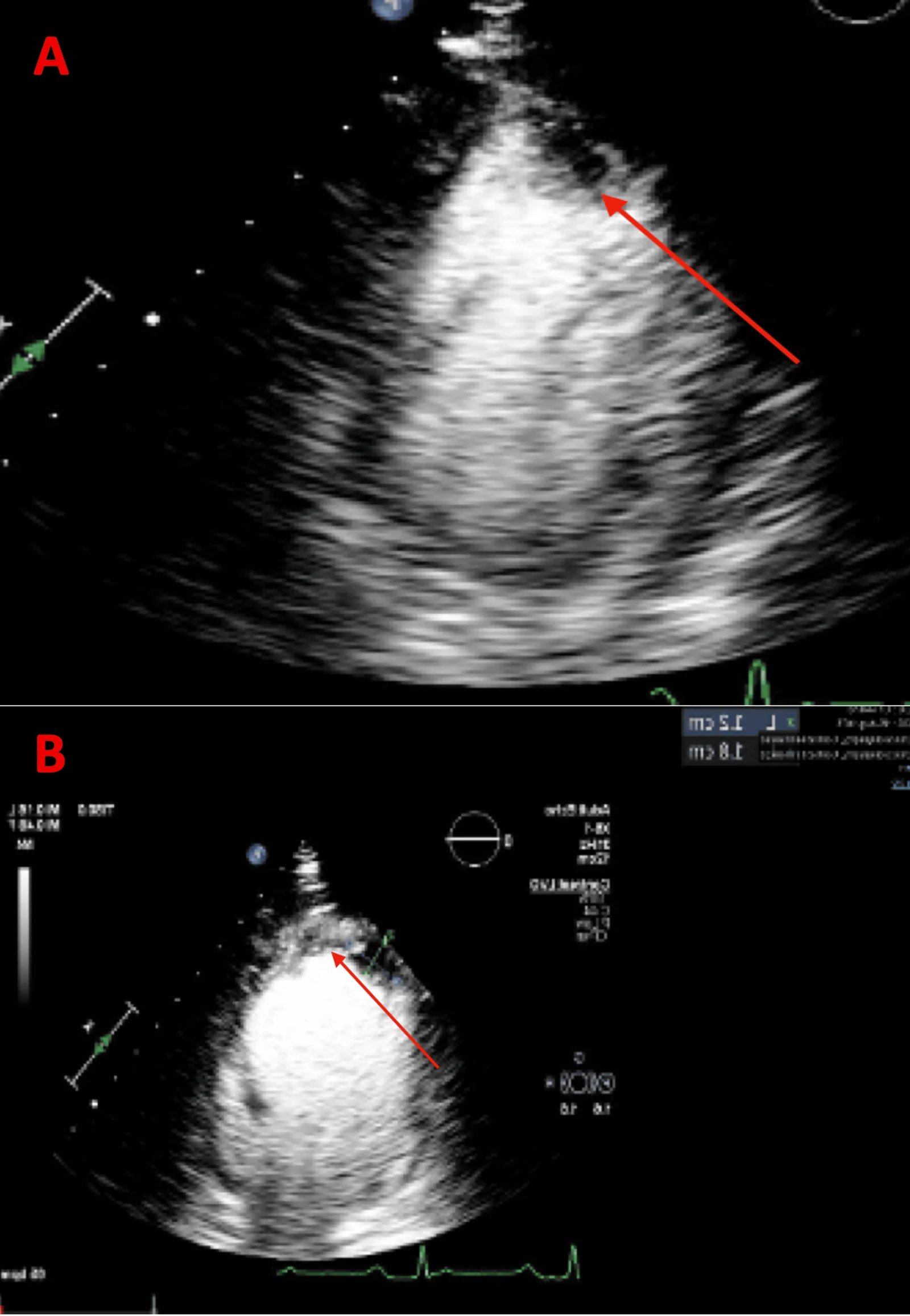
(A) and (B) show a left ventricular (LV) apical thrombus on contrast echocardiography (red arrows).

He underwent coronary angiography two days later. The procedure was initially attempted via the right radial artery; however, an incidental finding of total right subclavian artery occlusion was encountered, and the procedure was subsequently completed via the left radial artery. This showed the following findings (Figures 3-4): dominance: right dominant; left main stem (LMS): normal; left anterior descending (LAD) mid to distal segment: a long-segment dissection with diffuse luminal narrowing was noted, consistent with type 2 SCAD (variant 2A); left circumflex (LCX): distal LCX minor irregularities; right coronary artery (RCA) proximal to mid RCA: spiral dissection was noted, also consistent with Type 2 SCAD (variant 2A); right subclavian artery (RSA) is totally occluded. Thrombolysis in myocardial infarction (TIMI) flow grades were not documented in the catheterization report available for this case, and intravascular ultrasound (IVUS) or optical coherence tomography (OCT) was not performed during the diagnostic angiography; the diagnosis of SCAD was made based on the characteristic angiographic appearance. The diagnosis was SCAD in both RCA and LAD (type 2, variant 2A) and total occlusion of the RSA.

**Figure 3:**
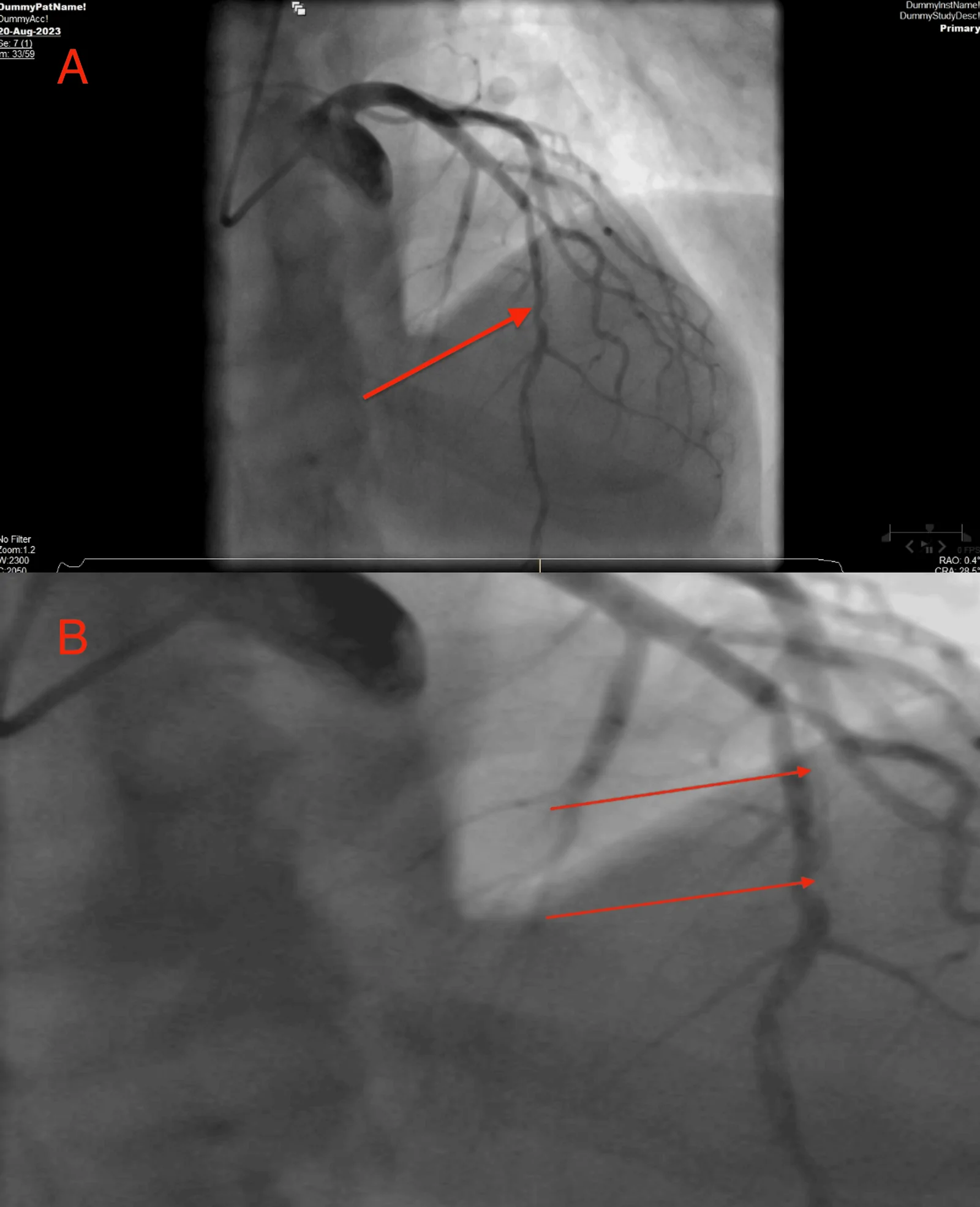
(A) Coronary angiography view of the left coronary tree demonstrating the LAD dissection (red arrow). (B) Additional image showing dissection starting from mid to distal LAD (red arrows), consistent with type 2 SCAD (variant 2A).

**Figure 4:**
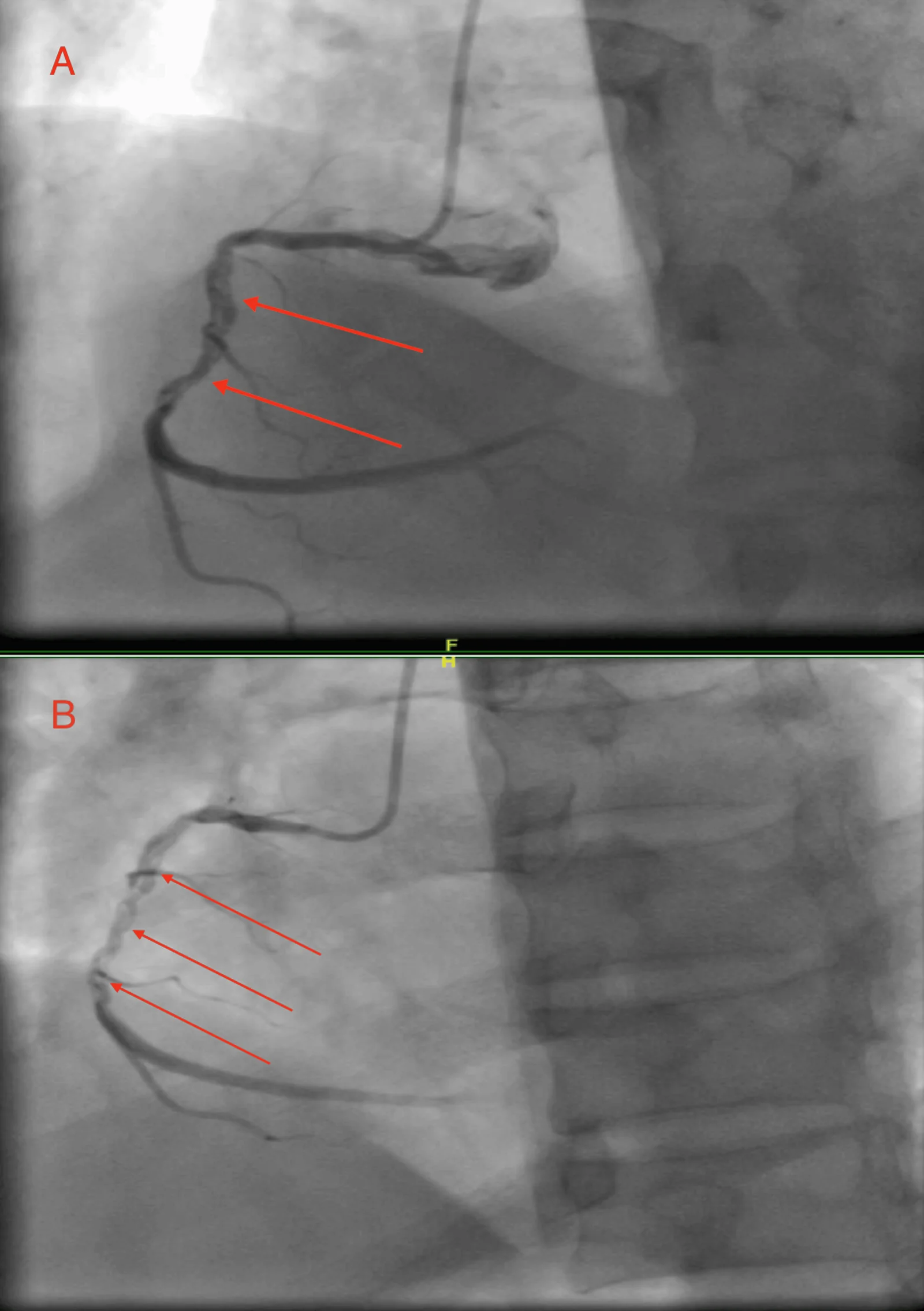
(A) and (B) show dissection of the ostial to mid right coronary artery on coronary angiography (red arrows), consistent with type 2 spontaneous coronary artery dissection (SCAD) (variant 2A).

With the finding of occluded RSA, he underwent a computed tomography (CT) aortogram (entire aorta) scan the following day, and this confirmed a complete luminal occlusion of the proximal 0.5 cm length segment of the RSA at its origin (likely old occlusion) with good caliber and flow of contrast noted distally and within its branches (proximal segments), representing subclavian steal syndrome changes (Figure 5). Otherwise, the rest of the CT aortic angiography study appeared unremarkable, with no wall thickening, luminal stenosis, corrugation, or aneurysmal dilatation of the aorta or its major branches; aortic root measurements were within normal limits (aortic annulus 2.3 cm, sinus of Valsalva 3.4 cm, sinotubular junction 2.7 cm, mid aortic arch 2.2 cm) (Figure 6). These findings did not fulfill imaging criteria for Takayasu arteritis.

**Figure 5:**
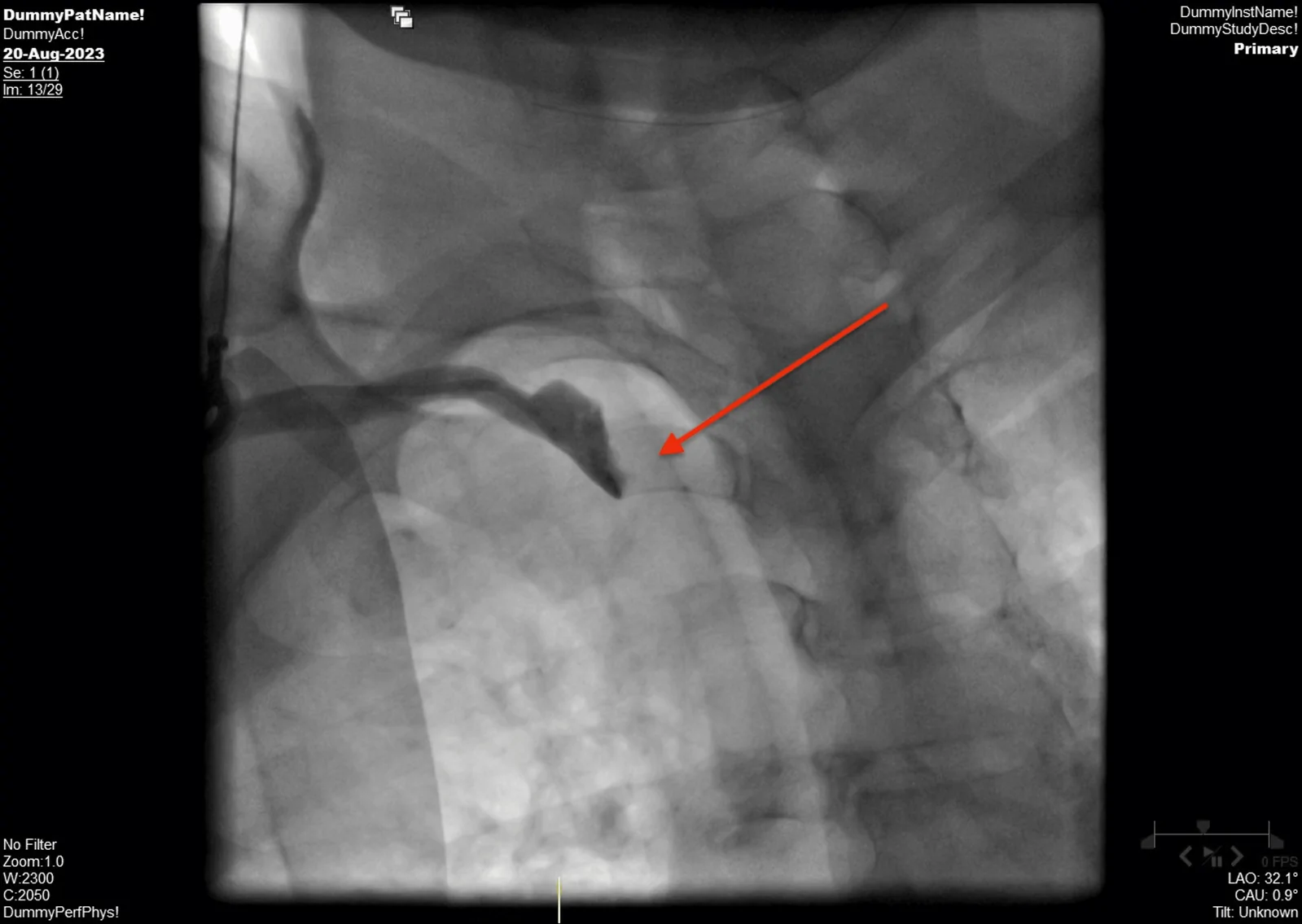
Angiographic image demonstrating the right subclavian artery occlusion (red arrow).

**Figure 6:**
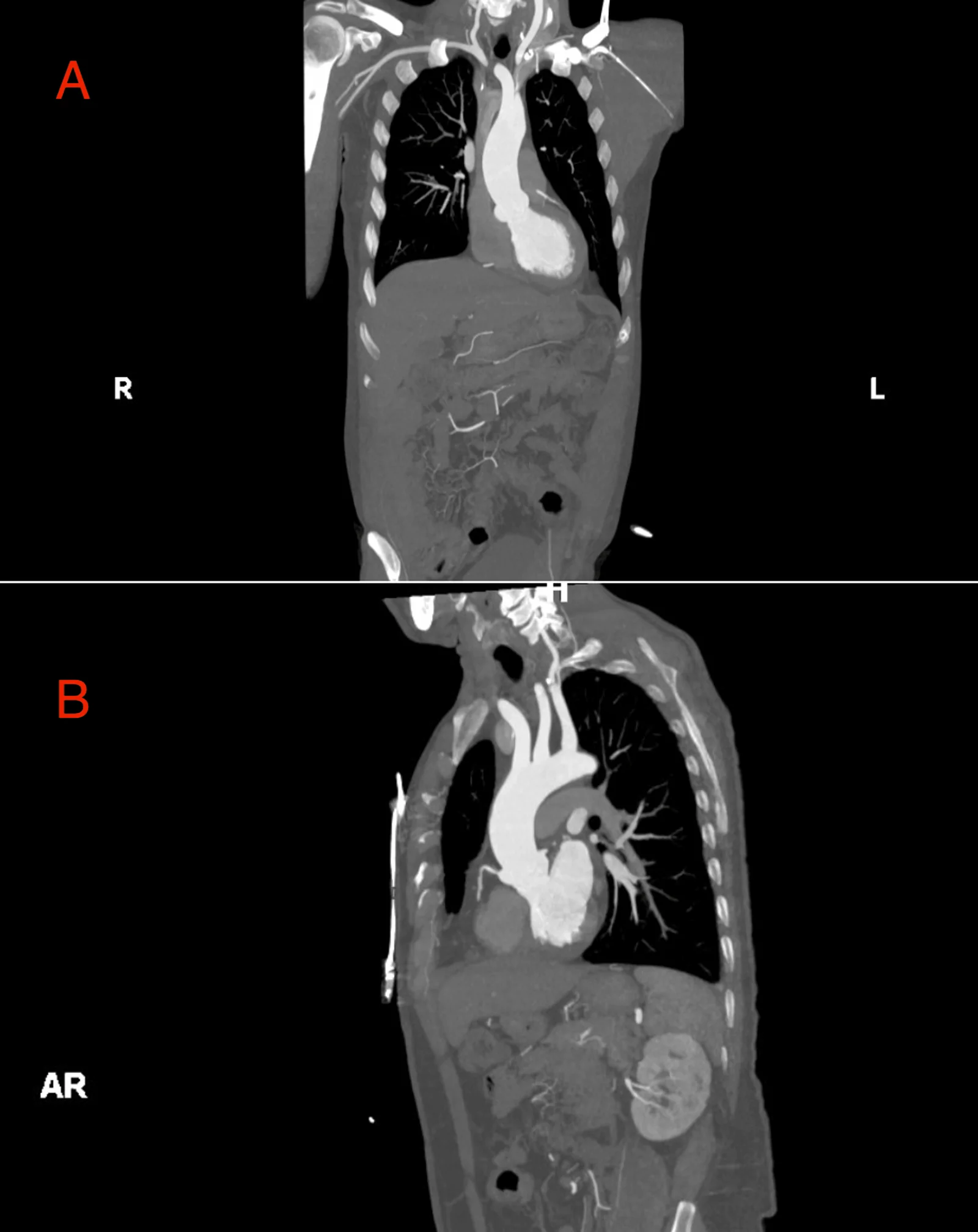
(A) CT aortogram, coronal reformat, demonstrating the aorta from the arch to the iliac bifurcation without aneurysmal dilatation, stenosis, or wall thickening. (B) CT aortogram, sagittal view, demonstrating the aortic root and arch.

At this stage, a diagnosis of vasculitis, including Takayasu arteritis, was considered. An autoimmune profile was ordered, which returned with normal anti-neutrophil cytoplasmic antibodies (ANCA), antinuclear antibodies (ANA), connective tissue disease screening panel (CTD), and complement components C3 and C4 levels. Inflammatory markers, including C-reactive protein (CRP) and erythrocyte sedimentation rate (ESR), were also within normal limits. No immunosuppressive or steroid therapy was initiated, as the autoimmune and inflammatory workup did not support an active vasculitic process. He was treated conservatively, with consultation of the vascular team, and was discharged 12 days after index admission with the following medications: clopidogrel 75 mg, warfarin 2 mg, atorvastatin 40 mg, empagliflozin 10 mg, bisoprolol 2.5 mg, pantoprazole 40 mg, sacubitril/valsartan (Entresto), and glyceryl trinitrate spray as needed.

During his hospital stay, the patient remained hemodynamically stable with no recurrence of chest pain, no documented arrhythmias, and no clinical evidence of heart failure decompensation. He did not require percutaneous coronary intervention (PCI) or surgical revascularization. He was discharged on day 12 with stable vital signs and clear outpatient follow-up arrangements.

The patient was followed in the outpatient clinic at two and four weeks after discharge for clinical review and INR management, reporting no recurrence of chest pain, dyspnea, syncope, or palpitations, and no bleeding complications related to anticoagulation. Repeat echocardiography was advised to reassess LV function and resolution of the apical thrombus; however, the patient did not undergo this study and subsequently missed his follow-up appointment, having returned to his home country. As a result, interval change in LV function and thrombus size could not be assessed, which represents a limitation of this case.

## Discussion

SCAD is a pathological process in which separation of the coronary arterial wall layers occurs, with or without an accompanying intimal tear, leading to the formation of a false lumen. The dissection typically originates approximately 2 cm from the coronary ostium and extends distally, either between the intimal and medial layers or between the media and adventitia [1].

SCAD most frequently affects younger individuals, especially peripartum or postpartum women who lack traditional cardiovascular risk factors, and the great majority of reported cases involve a single coronary vessel, typically the LAD. Multivessel involvement, as demonstrated in our case, is considerably less common, with only a limited number of cases reported [2].

Although multivessel SCAD, compared with single-vessel SCAD, has been linked to an increased risk of stroke both during hospitalization and on long-term follow-up, no significant differences were observed in clinical outcomes, including in-hospital major adverse events and major adverse cardiac or cerebrovascular events during follow-up [2].

In the majority of cases, an underlying predisposing arterial condition or identifiable cause is present, whereas up to 20% of cases are classified as idiopathic [4]. Notably, our patient had none of these classical predisposing factors - no connective tissue disorder, pregnancy history, mechanical or emotional stressors, or family history of SCAD - placing this case within that idiopathic subset.

A further notable feature of this case was the incidental finding of a chronic right subclavian artery occlusion alongside the multivessel coronary dissection. Associated arteriopathies, including fibromuscular dysplasia, are recognized in a substantial proportion of SCAD patients, and systemic inflammatory or autoimmune vasculitides such as Takayasu arteritis have also been described in association with coronary and peripheral arterial dissection [4]. Given this coexistence, an underlying vasculitic process was considered in our patient; however, CT aortography showed none of the wall thickening, luminal stenosis, corrugation, or aneurysmal change expected in active large-vessel vasculitis [6], and autoimmune and inflammatory markers (ANCA, ANA, connective tissue disease screen, complement levels, CRP, and ESR) were all within normal limits. These findings argue against Takayasu arteritis or another inflammatory arteriopathy and instead suggest that the subclavian occlusion represents a separate, likely chronic, incidental vascular process rather than a unifying etiology for the multivessel dissection. This distinction was clinically important, as it meant immunosuppressive therapy was not indicated and supported continued conservative management directed at the coronary and thromboembolic findings alone. To our knowledge, coexistent subclavian artery occlusion has only rarely been reported alongside multivessel SCAD, and this association may warrant further characterization in larger series.

The angiographic features of SCAD are categorized into three types [3]: Type 1: the classic pattern, showing multiple radiolucent lumens or contrast staining of the arterial wall. Type 2: characterized by diffuse narrowing of the artery, often exceeding 20 mm in length. In variant 2A, the narrowing is flanked by normal segments both proximally and distally to the intramural hematoma, whereas in variant 2B, the stenosis extends all the way to the distal end of the vessel. Type 3: resembles atherosclerotic disease, presenting as a focal or short tubular stenosis usually under 20 mm, which necessitates optical coherence tomography (OCT) or intravascular ultrasound (IVUS) to confirm the diagnosis.

In our patient, both the LAD and RCA dissections demonstrated diffuse, long-segment intramural hematoma bordered by normal-appearing vessel segments proximally and distally, without angiographic evidence of a double lumen or contrast staining typical of type 1; both lesions were therefore classified as type 2 SCAD, variant 2A.

Management of SCAD is guided primarily by the patient's clinical presentation, hemodynamic stability, the location and extent of the dissection, and the number of vessels involved, making individualized treatment essential [7].

For patients who are hemodynamically stable or have involvement of small vessels, conservative or medical management is usually recommended and is reported as the most commonly employed approach in case studies. Conversely, patients with hemodynamic instability or ongoing ischemia, particularly when the proximal segment of a coronary artery is severely affected, are better candidates for PCI or coronary artery bypass grafting (CABG). PCI can restore flow by either stent placement or balloon angioplasty in the narrowed segment, effectively sealing the dissection and relieving ischemia [8]. Advancing the coronary guidewire into the true lumen can be challenging, but intravascular imaging techniques such as IVUS and OCT assist in proper guidewire placement and accurate stent sizing. CABG is typically reserved for LMS dissections, failed PCI (TIMI 1-2 flow), or situations where PCI is technically unfeasible even at experienced centers [5]. In our patient, the absence of hemodynamic instability, recurrent ischemia, or proximal left main involvement supported the conservative strategy that was adopted.

The use of systemic anticoagulation in SCAD remains an area of ongoing debate. Because of theoretical concerns that anticoagulation may accentuate bleeding into the intramural hematoma and extend the dissection, expert guidance generally recommends discontinuing systemic anticoagulation once SCAD is diagnosed, unless there is an accompanying indication such as an intraluminal or left ventricular thrombus [3]. In our patient, therapeutic anticoagulation with heparin, followed by warfarin, was continued specifically to treat the large LV apical thrombus rather than the coronary dissection itself, consistent with this guidance.

Mortality rates in SCAD range from 1% to 5%, yet approximately 14% of patients require urgent in-hospital revascularization. In addition, 2.2-7.4% of patients may need CABG following initial medical therapy or PCI [4]. Our patient's uncomplicated hospital course without recurrent ischemia or need for revascularization is consistent with this generally favorable prognosis when conservative management is appropriate.

## Conclusions

SCAD is an uncommon but important cause of chest pain and ACS, particularly in young patients without traditional cardiovascular risk factors. Multivessel involvement is even less frequent, reported in approximately 9-23% of cases, and may be associated with underlying conditions, such as vasculitis; in our patient, however, CT aortography did not meet imaging criteria for Takayasu arteritis, and the case is best characterized as idiopathic multivessel SCAD. Management strategies must be individualized, with conservative medical therapy being the preferred approach for stable patients, while PCI or CABG may be necessary in cases with ongoing ischemia, hemodynamic compromise, or proximal vessel involvement. Our case demonstrates that carefully selected patients with multivessel SCAD can be successfully managed conservatively, achieving favorable clinical outcomes.

This highlights the importance of early recognition, appropriate risk stratification, and individualized treatment planning in improving patient prognosis in SCAD.
